# Neural network modeling of altered facial expression recognition in autism spectrum disorders based on predictive processing framework

**DOI:** 10.1038/s41598-021-94067-x

**Published:** 2021-07-26

**Authors:** Yuta Takahashi, Shingo Murata, Hayato Idei, Hiroaki Tomita, Yuichi Yamashita

**Affiliations:** 1 Department of Psychiatry, Tohoku University Hospital, Sendai, Japan; 2 Department of Information Medicine, National Center of Neurology and Psychiatry, 4-1-1 Ogawa-Higashi, Kodaira, Tokyo 187-8502 Japan; 3 Department of Electronics and Electrical Engineering, Faculty of Science and Technology, Keio University, Tokyo, Japan; 4 Department of Intermedia Studies, Waseda University, Tokyo, Japan

**Keywords:** Computational models, Network models, Autism spectrum disorders

## Abstract

The mechanism underlying the emergence of emotional categories from visual facial expression information during the developmental process is largely unknown. Therefore, this study proposes a system-level explanation for understanding the facial emotion recognition process and its alteration in autism spectrum disorder (ASD) from the perspective of predictive processing theory. Predictive processing for facial emotion recognition was implemented as a hierarchical recurrent neural network (RNN). The RNNs were trained to predict the dynamic changes of facial expression movies for six basic emotions without explicit emotion labels as a developmental learning process, and were evaluated by the performance of recognizing unseen facial expressions for the test phase. In addition, the causal relationship between the network characteristics assumed in ASD and ASD-like cognition was investigated. After the developmental learning process, emotional clusters emerged in the natural course of self-organization in higher-level neurons, even though emotional labels were not explicitly instructed. In addition, the network successfully recognized unseen test facial sequences by adjusting higher-level activity through the process of minimizing precision-weighted prediction error. In contrast, the network simulating altered intrinsic neural excitability demonstrated reduced generalization capability and impaired emotional clustering in higher-level neurons. Consistent with previous findings from human behavioral studies, an excessive precision estimation of noisy details underlies this ASD-like cognition. These results support the idea that impaired facial emotion recognition in ASD can be explained by altered predictive processing, and provide possible insight for investigating the neurophysiological basis of affective contact.

## Introduction

Impaired affective contact is a core symptom of autism spectrum disorder (ASD), as reported by Kanner^1^, for which the recognition of facial emotion is an essential skill^2^. In fact, a previous meta-analysis found that individuals with ASD have difficulty in facial emotion recognition^3^, and a substantial number of studies have reported atypical processing of facial stimuli or difficulties with real-life emotional recognition in ASD^4^. The neural basis of facial emotion recognition has been intensively investigated by functional neuroimaging studies in healthy subjects^5–7^. These previous studies have revealed a hierarchical structure among several brain regions; namely, the activities in the visual cortex correspond to the processing of lower-level sensory information, including features of faces, and the activity patterns in higher-level brain areas such as the fusiform gyrus or superior temporal sulcus correspond to the emotion category^6, 7^. In addition, clinical studies have demonstrated that the activity pattern in the facial emotion recognition network is altered in ASD^4, 8^. Despite these findings regarding the anatomical neural networks related to facial emotion expression, the developmental process responsible for categorizing visual facial expression information into emotional groups and its alteration in ASD are largely unknown. Although the emergence of emotional categories was hypothesized to be a natural process based on the similarity among facial feature patterns^9, 10^, this hypothesis and its alterations in ASD have rarely been investigated regarding information processing in actual neural systems. Computational psychiatry is an emerging field for investigating information processing in the brain, which aims to bridge the gap between the respective biological findings and psychiatric symptoms^11^. Therefore, the current study aims to understand the facial emotion recognition process and its impairment in ASD through a computational approach.

Predictive processing (or predictive coding) is a well-studied cognitive framework in computational psychiatry^12^. Predictive processing explains perceptual and cognitive processes as the interactions between the top-down prediction (i.e., prediction for the next sensory input using the prior beliefs and current sensory signals) and bottom-up modulation (i.e., the updates of prior beliefs based on the prediction error) through the computational principle of minimizing prediction error. Predictive processing theory suggests that various brain functions, such as perception, cognition, and behaviors, can be understood by minimizing prediction error^12^. Additionally, estimation of the precision of sensory information plays a key role in prediction error minimization during predictive processing, in the sense that the estimation of sensory precision regulates the degree of updates of prior beliefs. Therefore, an inappropriate estimation of sensory precision may result in alterations in perceptions, cognitions, and behaviors. Indeed, there exists several conceptual theories attributing the characteristic perception and cognition deficits in ASD to the inappropriate precision in predictive processing, referred to as “aberrant precision theory”^13–15^. According to the aberrant precision theory, while normally developed brains tolerate a certain degree of prediction error in extracting abstract information from noisy sensory information, the ASD brain is hypothesized to aberrantly estimate precision on the sensory input, and the resulting inappropriate renewals in prior beliefs make it difficult to abstract higher-level information. Aberrant precision theory can explain multiple domains of ASD symptoms such as cognition, perception, sensorimotor abilities, and social functioning^14^. A substantial number of behavioral and psychological studies have investigated the aberrant precision theory^16–18^, and the degree of altered precision has been shown to be correlated with the severity of some ASD symptoms^18, 19^. However, the impaired affective contact of core symptoms in ASD has not yet been investigated from the viewpoint of aberrant precision theory. Therefore, the current study investigated the relationship between the alteration in emotional recognition in ASD and the estimation of sensory precision using computational psychiatry methods.

Facial emotion recognition is a dynamic process based on visual contextual information^20^, which can be modeled using a predictive processing framework. To investigate the dynamic sensory information processing of facial emotion recognition, the current study utilized a stochastic continuous time recurrent neural network with parametric bias (S-CTRNNPB). The S-CTRNNPB models system-level information processing in the biological brain and can implement predictive processing by hierarchical networks integrating top-down prediction and bottom-up modulation^21, 22^. The higher-level neural representation in S-CTRNNPB is clustered based on the similarity among the sensory inputs, which is comparable to emotional category acquisition from facial expressions. Furthermore, unlike simple recurrent neural networks, the S-CTRNNPB can estimate the precision of sensory information; then, the S-CTRNNPB succeeds in simulating autistic behavior based on aberrant precision theory^23, 24^. Based on these findings, the current study utilized the S-CTRNNPB to model facial emotion recognition based on a predictive processing framework.

Considering the abovementioned findings, we hypothesized the following: First, emotional categories would emerge in the natural course of self-organization in higher-level neurons during the developmental learning process based on a predictive processing framework, even though emotional labels are not explicitly instructed. Second, the cognitive process to estimate the emotion of unseen facial expressions can be understood as the process of adjusting higher-level neural states by minimizing the prediction error. Third, altered facial emotion recognition in ASD can be understood as altered predictive processing. To investigate these hypotheses, facial expression recognition was modeled through predictive processing, in which the S-CTRNNPB was trained to predict the dynamic changes of facial features in the movies of facial expression through prediction error minimization. In the test phase, the network was tested to see if unseen sequences of facial expressions could be predicted through the process of minimizing the prediction error. Self-organized higher-level neuron representation was evaluated in terms of the clustering of emotion categories. Finally, we evaluated the influence of the alternations in the network characteristics on the aberrant estimation of sensory precision and its ASD-like cognition.

## Methods and materials

### Facial expression movie datasets and preprocessing

The facial expression movies were obtained from the CK+ public database^25, 26^, which included movies in which the face changed from neutral to peak emotions. Written informed consent was obtained for analysis and publication of the images. The movies consisted of image frames that were taken 30 times per second. Each movie in the CK+ database was labeled based on criteria regarding the movements of facial landmarks (i.e., facial action coding system)^27^ and the perceptual judgments from multiple testers^25, 26^. The current study used the movies focused on the basic six emotions (Anger, Disgust, Fear, Happiness, Sadness, and Surprise)^2^. In the CK+ database, only a few of the total six emotional expressions are included for most subjects. However, an imbalance in the number of emotions in training and test data would hinder the investigation of the relationship among the higher-level neuronal representations of the six emotions. Therefore, we used eightfold cross-validation with an equal number of emotions in each group to evaluate the model (see Supplementary Methods for details).

**Figure 1:**
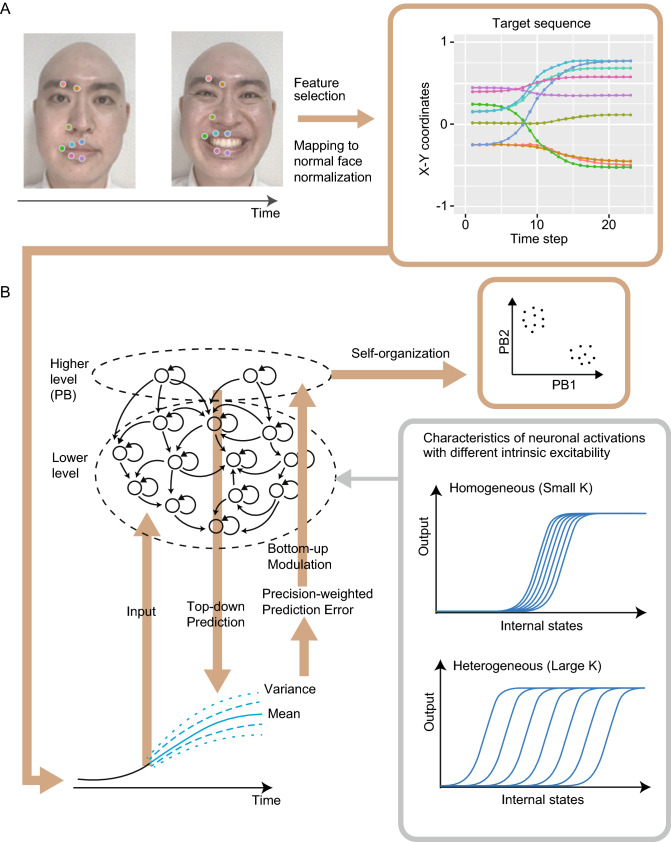
(**A**) The preprocessing of facial expression movies. The trajectories of the 9 facial features corresponding to the facial expressions were utilized as target sequences after mapping to normal face normalization, which is detailed in the methods and materials section. The face image used in this conceptual diagram is that of one of the authors (YT). (**B**) Overview of S-CTRNNPB. The S-CTRNNPB is a hierarchical recurrent neural network model implementing top-down prediction and bottom-up modulation processes, aiming to minimize precision-weighted prediction error. To simulate autistic cognition, we manipulated the heterogeneity of intrinsic neural excitability by changing K parameters (i.e., the variance of the activity thresholds in lower-level neurons). It is noteworthy that the emotional labels were not provided to the S-CTRNNPB in the learning process, and higher-level neuronal representations were self-organized based on the similarity among the sensory inputs. *S-CTRNNPB* stochastic continuous time recurrent neural network with parametric bias, *PB* parametric bias.

From the movie, we extracted the X–Y coordinates of 68 facial landmarks (136 features) using automatic face detection and feature tracking system^25^. Thereafter, owing to the limitation of computational cost, features with very strong correlations with other features and almost immobile features were removed; the remaining nine features were used for subsequent analysis (i.e., the X-coordinate of the lip corner, and Y-coordinates of the middle of the eyebrow, the inner eyebrow, ala of the nose, the central upper lip, upper lip vermillion, lip corner, the central lower lip and lower lip vermillion, in the right face). See also Fig. 1A and Supplementary Methods.

The preprocessing of the sequence data was performed as follows. Suppose that we have a sequence of ${{\varvec{x}}}^{({\varvec{i}},{\varvec{j}})}=({x}_{1}^{\left(i,j\right)},{x}_{2}^{\left(i,j\right)},{x}_{3}^{\left(i,j\right)},\ldots ,{x}_{{T}^{(i)}}^{\left(i,j\right)})$ of the $j$th feature ($1\le j\le 9$) in the $i$th facial expression sequence ($1\le i\le 96$), which has ${T}^{(i)}$ time steps. First, from the vector of sequence, its first step value is subtracted to make each feature’s first step value zero.

1 $${{{\varvec{x}}}^{\boldsymbol{^{\prime}}}}^{\left({\varvec{i}},{\varvec{j}}\right)}=\left(0,{x}_{2}^{\left(i,j\right)}-{x}_{1}^{\left(i,j\right)},{x}_{3}^{\left(i,j\right)}-{x}_{1}^{\left(i,j\right)},\ldots ,{x}_{{T}^{\left(i\right)}}^{\left(i,j\right)}-{x}_{1}^{\left(i,j\right)}\right).$$

Next, the values were scaled to a range of values into [− 0.9, 0.9] for each feature over all target sequences.

2 $$MAX=\mathit{max}\left({max}_{1\le t\le {T}^{\left(1\right)}}\left({{x}^{^{\prime}}}_{t}^{\left(1,j\right)}\right), { max}_{1\le t\le {T}^{\left(2\right)}}\left({{x}^{^{\prime}}}_{t}^{\left(2,j\right)}\right), \ldots ,{ max}_{1\le t\le {T}^{\left(96\right)}}\left({{x}^{^{\prime}}}_{t}^{\left(96,j\right)}\right) \right),$$

3 $$MIN=\mathit{min}\left({min}_{1\le t\le {T}^{\left(1\right)}}\left({{x}^{^{\prime}}}_{t}^{\left(1,j\right)}\right), { min}_{1\le t\le {T}^{\left(2\right)}}\left({{x}^{^{\prime}}}_{t}^{\left(2,j\right)}\right), \ldots ,{ min}_{1\le t\le {T}^{\left(96\right)}}\left({{x}^{^{\prime}}}_{t}^{\left(96,j\right)}\right) \right),$$

4 $${{x}^{^{\prime\prime} }}^{\left(i,j\right)}=\frac{{{x}^{^{\prime}}}^{\left(i,j\right)}-MIN}{MAX-MIN}\times 1.8-0.9.$$

The subtraction in Eq. (1) was referred to as “mapping to normal face normalization” because this process unifies the positions of features in the first step among all the target sequence. The resulting sequence data are referred to as “target sequences” and subjected to the following analysis.

### Neural network model

In the current study, the main component of the facial emotion recognition process based on the predictive processing framework was modeled using S-CTRNNPB (Fig. 1B)^21, 22^. This framework explains cognition through two processes: (i) top-down prediction of the next sensory input based on the current sensory input and the internal states of the network (i.e., prior belief) and (ii) bottom-up modulation based on precision-weighted prediction error minimization. While the lower-level neurons in S-CTRNNPB represent the dynamics (i.e., short-term sensory processing) of the target sequence, the higher-level neurons represent the abstract meaning for all steps of each sequence. The higher-level neurons in the S-CTRNNPB model are referred to as the parametric bias (PB). The input to the S-CTRNNPB are sensory states corresponding to the facial image at the current time step, and the S-CTRNNPB outputs the prediction of sensory states for the next time step corresponding to the changes in facial images. As a result, S-CTRNNPB can generate a sequence corresponding to the dynamic facial expression of a particular emotion (i.e., target sequence). The S-CTRNNPB predicts not only the value of the target sequence in the next step, but also the precision (i.e., variance of the target values assuming a normal distribution including noise).

In the top-down prediction of S-CTRNNPB, the internal state of the $\text{i}$th neuron at time step t was calculated as

5 $$u_{{t,i}}^{{\left( s \right)}} = \left\{ {\begin{array}{*{20}l} {u_{{t - 1,i}}^{{\left( s \right)}} } \hfill & {i \in I_{P} } \hfill \\ {\frac{1}{{\tau _{i} }}\left( {\sum\limits_{{j \in I_{I} }} {w_{{ij}} } x_{{t,j}}^{{\left( s \right)}} + \sum\limits_{{j \in I_{L} }} {w_{{ij}} } l_{{t - 1,j}}^{{\left( s \right)}} + \sum\limits_{{j \in I_{P} }} {w_{{ij}} } p_{{t,j}}^{{\left( s \right)}} + a_{i} } \right) + \left( {1 - \frac{1}{{\tau _{i} }}} \right)u_{{t - 1,i}}^{{\left( s \right)}} } \hfill & {i \in I_{L} } \hfill \\ {\sum\limits_{{j \in I_{L} }} {w_{{ij}} } l_{{t,j}}^{{\left( s \right)}} + a_{i} } \hfill & {i \in I_{M} ,I_{V} } \hfill \\ \end{array} } \right.,$$

where *I*_*P*_*, I*_*L*_, *I*_*I*_, *I*_*M*_, and *I*_*V*_ are the index sets of the PB, lower-level, input, predicted mean, and estimated variance neurons, respectively; *w*_*ij*_ is the synaptic connection weight from the *j*th neuron to the *i*th neuron; ${x}_{t,j}^{\left(s\right)}$ is the jth external input value at time step $t$ of the $\text{s}$th sequence; ${l}_{t,j}^{\left(s\right)}$ is the jth lower-level neuron activity; ${p}_{t,j}^{\left(s\right)}$ is the jth PB activity; ${\tau }_{i}$ is the time constant of the $\text{i}$th neuron; and ${a}_{i}$ is the activity threshold of the $i$th neuron.

The output of each neuron is calculated by the activation function as shown below.

6 $${p}_{t,i}^{\left(s\right)}={\text{tanh}}\left({u}_{t,i}^{\left(s\right)}\right) 1\le t\cap i\in {I}_{P},$$

7 $${l}_{t,i}^{\left(s\right)}={\text{tanh}}\left({u}_{t,i}^{\left(s\right)}\right) 0\le t\cap i\in {I}_{L},$$

8 $${y}_{t,i}^{\left(s\right)}={\text{tanh}}\left({u}_{t,i}^{\left(s\right)}\right) 1\le t\cap i\in {I}_{M},$$

9 $${v}_{t,i}^{\left(s\right)}={\text{exp}}\left({u}_{t,i}^{\left(s\right)}\right) 1\le t\cap i\in {I}_{V}.$$

Bottom-up modulations are the process of parameter optimization based on precision-weighted prediction error minimization. This process aims to minimize the following negative log-likelihood:

10 $${L}_{t,i}^{\left(s\right)}=\frac{\text{ln}\left(2\pi {v}_{t,i}^{\left(s\right)}\right)}{2}+\frac{{\left({\widehat{y}}_{t,i}^{\left(s\right)}-{y}_{t,i}^{\left(s\right)}\right)}^{2}}{2{v}_{t,i}^{\left(s\right)}},$$

where ${\widehat{y}}_{t,i}^{\left(s\right)}$ is the target input value. Parameter optimization is performed by minimizing the sum of the negative log-likelihood over all feature dimensions, time steps, and sequences using the gradient descent method^28^. Top-down prediction and bottom-up modulation are detailed in the Supplementary Methods.

### Experimental procedure

The experimental procedure consisted of training and test phases. The training phase is analogous to the developmental learning process and aims to optimize the network structure (i.e., synaptic weights) of the S-CTRNNPB and the PB activity associated with each target sequence. It is noteworthy that the network was trained only for predicting the changes in the sensory states of facial expression movies, but the labels of emotions were not provided to the model. After the training, each target sequence was associated with particular activities of PBs, and the relationships among the target sequences (similarity and differences) were expected to be “self-organized” in the state-space of PB activities.

In the test phase, the network was required to predict an unseen target sequence. In this test phase, while the network structure was fixed, the PB activities were updated to minimize the precision-weighted prediction error for an unseen test sequence. This PB update process for an unseen test sequence was regarded as “emotion recognition” based on the similarity of the PB activity for a test sequence to the PB clusters for the training sequences of a particular emotion category. A detailed explanation of the clustering index for PB spaces is provided in the Supplementary Methods section.

### Parameter manipulations to simulate autistic cognition

We simulated two pathological conditions of network structures based on biological or computational findings in ASD. First, the intrinsic heterogeneity of network excitability is important for efficient information processing^29–31^, and its alterations have been suggested to be related to ASD, i.e., altered “excitatory-inhibitory balance”^24^. In the current experiment, as shown in Eq. (11), the activity threshold of lower-level neurons (i.e., ${a}_{i}$ in Eq. (5)) is initialized to follow a Gaussian distribution and fixed without being updated by learning.

11 $${a}_{i}\sim \text{N}\left(0, K\right) \qquad K=0.001, 1, 1000,$$

The K parameter in Eq. (11) determines the heterogeneity of intrinsic neuronal excitability, and as the parameter K is increased, the excitability of the network becomes more heterogeneous. In the current analysis, the heterogeneous network with K = 1000 was regarded as a typical developmental model, and homogeneous networks with K = 0.001 and 1 were assumed to be possible ASD-like models. Second, as there are biological studies that showed that the brains of individuals with ASD have a greater number of cortical minicolumns (i.e., structures that constitute basic functional assemblies of neurons)^32, 33^, the influence of an increased number of lower-level neurons on the performance of the models was also evaluated in this study. To investigate the influences of the parameter K and the number of lower-level neurons on the model performance, the four representative network structures in Table 1 were subjected to the analysis described earlier.

**Table 1:** The representative neural network models for experiment.

	Excessively homogeneous network	Modestly homogeneous network	Heterogeneous network (typical development model)	Large network
K parameter^a^	0.001	1	1000	1000
The number of PBs	2	2	2	2
The number of lower-level neurons	500	500	500	1000

*PB* parametric bias.

^a^K parameter is the variance in activity thresholds in lower-level neurons (Eq. (5) in the Supplementary Methods), which determines the intrinsic heterogeneity of network excitability. When the parameter K is larger, the network excitability becomes more heterogeneous.

## Results

### The performance of neural networks of typical development model

**Figure 2:**
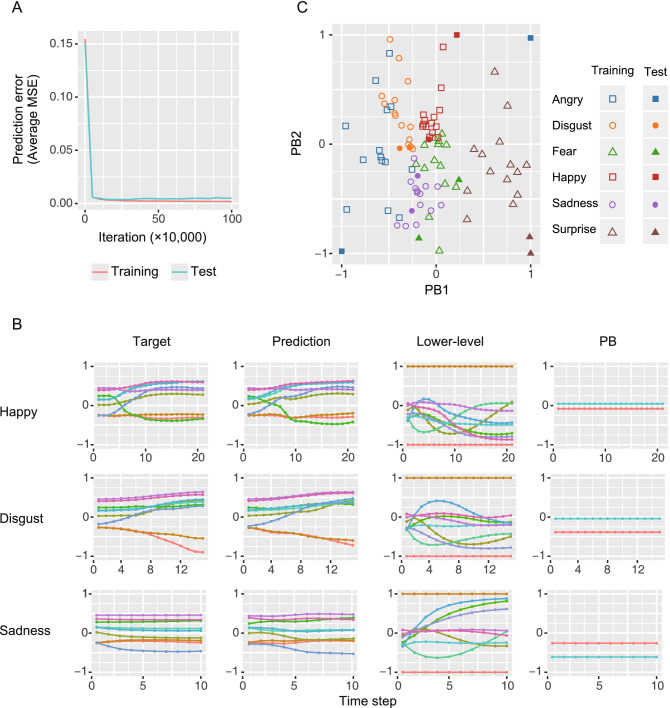
(**A**) The learning curves. The X axis indicates the number of iterations in the training process. The Y axis indicates the prediction error (MSE), which is averaged over the sequence length, feature numbers, sequence numbers, and eightfold cross-validation. Both the prediction error for training sequences as well as the prediction error for test sequences are shown in this figure. (**B**) Examples of target sequences, predicted sequences, lower-level neuron activities and PB activities for the test target sequences. For lower-level neuron activity, 10 representative neurons among 500 neurons are shown. (**C**) The PB activity representation. The color indicates emotional categories. The outlined symbols express the PBs corresponding to the training sequences, while the filled symbols express those to the test sequences. *MSE* mean squared error, *PB* parametric bias.

The learning curves indicated that both the prediction errors for the training and test sequences substantially decreased (Fig. 2A), suggesting that the model not only succeeded in reproducing the training target sequences, but also acquired the generalization capability to predict the unseen test target sequences.

Examples of the target and predicted sequences and the activities of lower-level neurons and PBs for the test target sequences are shown in Fig. 2B. The target sequences, which differed according to emotions, were well reproduced by network prediction. The activity pattern in the lower-level neurons corresponded to the short-term dynamics of the target sequences. Among the lower-level neurons, the activities of several neurons changed over time, while the activities of other neurons maintained almost constant levels of activity. On the other hand, the activity patterns of PBs seemed to correspond to a more abstract level of characteristics of a sequence independent of the short-term changes in sensory states (Fig. 2B).

The PB activities for all target sequences are illustrated in Fig. 2C. In Fig. 2C, the PBs corresponding to the training target sequences (outline symbols) seemed to be clustered according to the emotional categories. Based on this finding, although the emotion labels were not provided in the training, the emotional clusters successfully emerged through the predictive processing framework. In addition, the PB activities for the test sequences (filled symbols) that were optimized through the emotion recognition process by minimizing the precision-weighted prediction error were located close to the PB clusters for the training sequences of the same emotion (Fig. 2C), indicating that the models successfully recognized facial expressions of unseen test sequences.

### Predictive performances among various models

**Figure 3:**
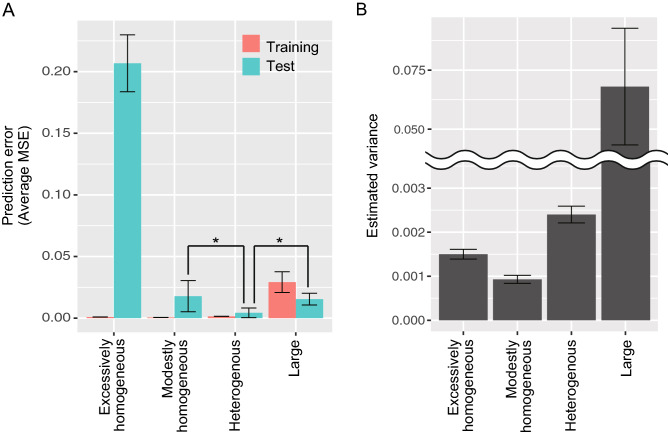
(**A**) Prediction error (average MSE) in various network models. The X axis indicates the network structures (detailed in Table 1) and the Y axis indicates the MSEs which are averaged over the sequence length, feature numbers, and sequence numbers. An enlarged view is shown in Supplementary Fig. S1A, and the results of various other networks are shown in Supplementary Figs. S1B, S2A. (**B**) Estimated variance in various network models. The estimated variances are averaged over the sequence length, feature numbers, and sequence numbers. *MSE* mean squared error.

The influence of the network characteristics on predictive processing was investigated using various network structures, including the intrinsic heterogeneity of network excitability and the size of the network (Table 1). Compared with the heterogeneous (typical development) network model, the excessively or modestly homogeneous network models had smaller training errors and larger test errors, while the network with an increased number of neurons (large network model) showed larger training and test errors (Fig. 3A; see Supplementary Figs. S1A,B, S2A for more details).

In relation to the aberrant precision hypothesis in ASD, the estimated variance of the target values was also investigated among the various models. Compared to the heterogeneous (typical development) network, the homogeneous network models tended to estimate the lower variance (i.e., higher precision) and the large network estimated the larger variance (i.e., lower precision) (Fig. 3B, see Supplementary Fig. S2B for more details). Based on the abovementioned findings, the excessively or modestly homogeneous network models estimated excessive precision on sensory input, resulting in overfitting (i.e., low training error and high test error), which is consistent with the ASD-like cognition based on the aberrant precision hypothesis.

### Emotion recognition among various models

**Figure 4:**
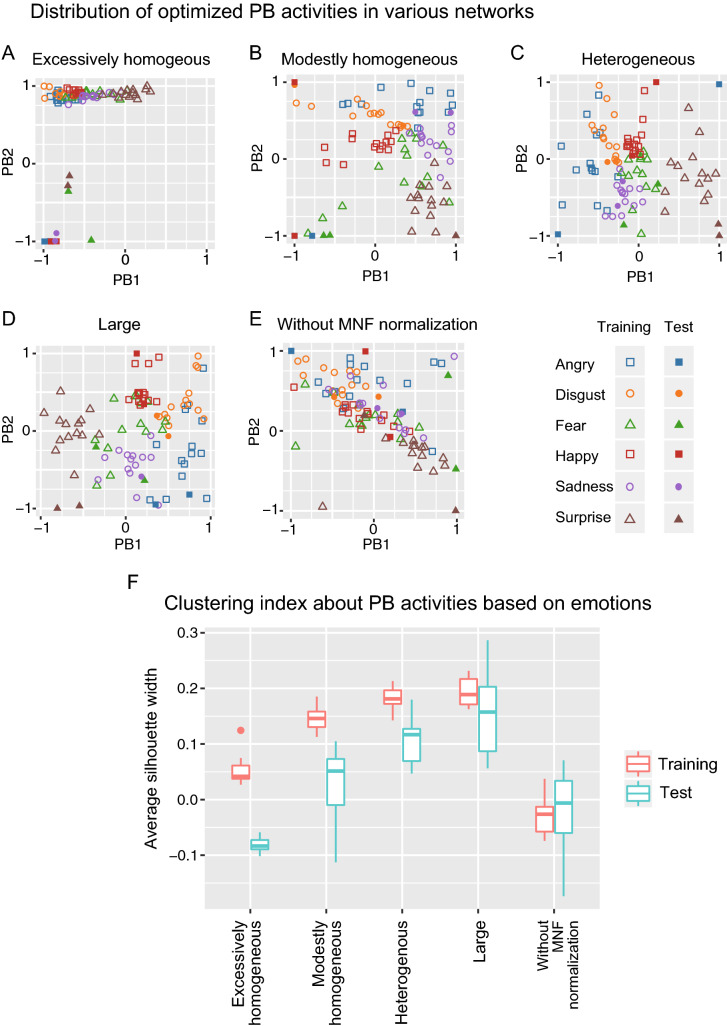
(**A–E**) The optimized PB activities in various networks. The color indicates emotional categories. The outlined symbols express the PBs corresponding to the training sequences, while the filled symbols express those to the test sequences. (**F**) The clustering index of the PB activities based on emotions. The X axis indicates the network models (Table 1) and the Y axis indicates average silhouette widths based on eightfold cross-validation. The silhouette width is a measure of similarity of an object to its own cluster compared to other clusters (detailed in Supplementary Methods). *PB* parametric bias, *MNF* normalization; mapping to normal face normalization.

Emotion recognition (i.e., the degree of clustering of PB activities for training sequences and optimized PB activities for unseen test sequences) was also compared among the various networks. For each model condition, the distribution of PB activities acquired for training and test datasets is illustrated in Fig. 4A–E, and the clustering index for these models based on the eightfold cross-validation is shown in Fig. 4F. In the excessively homogeneous network (Fig. 4A), the PB activities for training sequences were not clustered, and those for the test sequences were remotely located from those for the training sequences. Therefore, the excessively homogenous network model failed to acquire the PB representation corresponding to the emotional categories or recognize the similarity between training and test sequences within the same emotion. The clustering index showed that not only excessively but also modestly homogeneous networks showed a tendency toward weaker clustering for the training and test sequences (Fig. 4F). On the other hand, in the heterogeneous network (Fig. 4C,F) and large network (Fig. 4D,F), PB activity for training and test sequences were successfully clustered according to emotion.

Additionally, to evaluate the effect of mapping to normal face normalization on emotion recognition, the PB activity optimized for the target sequences in the heterogeneous network without this normalization are shown in Fig. 4E,F. Remarkably, without this normalization, the PB activities for training sequences were not clustered and those for the test sequences were remotely located from those for training sequences, suggesting that the process of mapping to a normal face was essential for the emergence of emotional categories and facial emotion recognition in the predictive processing framework.

### Tolerance of higher-level neural representation

**Figure 5:**
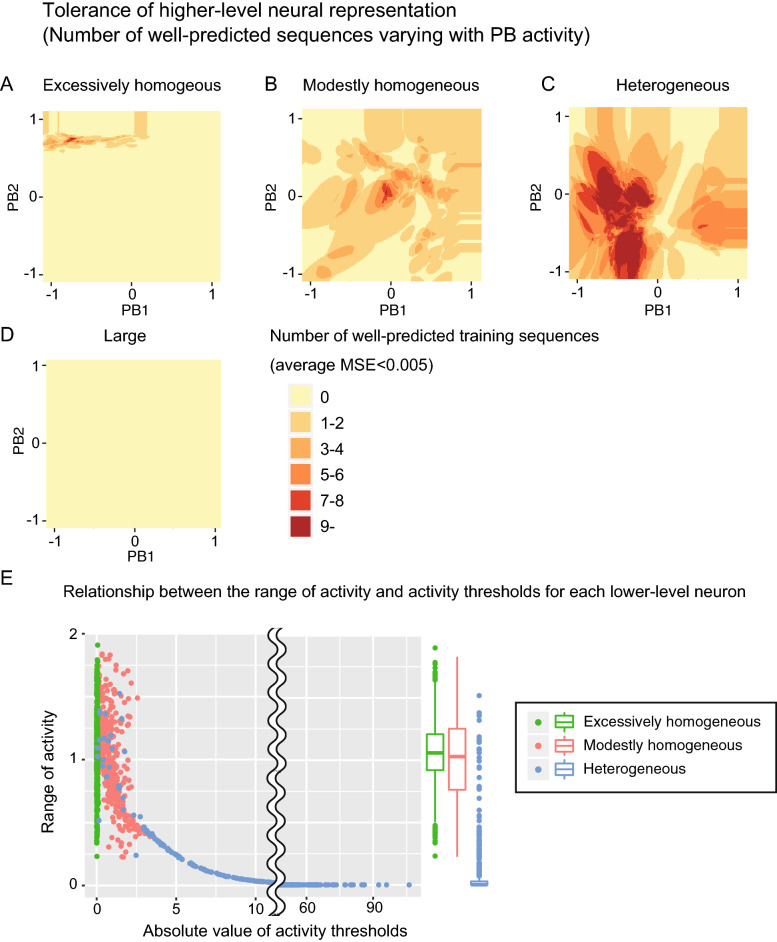
(**A–D**) Tolerance of higher-level neural representations. The heat maps show the numbers of well-predicted training sequences (i.e., average MSE < 0.005) varying with the PB activities. (**E**) Relationship between the range of activity and activity thresholds for each lower-level neuron. The X axis indicates the absolute values of activity thresholds for each lower-level neuron. The Y-axis indicates the range of activity (i.e., difference between the maximum and minimum outputs) for each lower-level neuron. The boxplots shows the distribution about the range of activity of lower-level neurons. *PB* parametric bias, *MSE* mean squared error. The figures were created using R software^45^ and the ggplot2 library^46^.

As mentioned above, the heterogeneous (typical development) network acquired the generalization capability to predict test target sequences using PB activity similar to those for training datasets with the same emotion. We then hypothesized that the PB representations in a heterogeneous network would tolerate subtle differences among the target sequences for prediction, while the homogeneous network would be fragile to subtle differences due to overfitting to a particular sequence of the training targets. To investigate this hypothesis, we evaluated the number of training sequences that could be predicted well (i.e., average mean squared error < 0.005) by changing the levels of PB activities for various network conditions. This number of successfully predicted sequences would reflect the tolerability of PB representation for application to different sequences. Figure 5A–D shows the number of well-predicted sequences for each PB activity with various network conditions in Table 1. Excessively or modestly homogeneous networks reproduced a smaller number of target sequences for each PB activity, which implies that the homogeneous network cannot tolerate the subtle difference among the target sequences even in the same emotion category (Fig. 5A,B). This intolerance to the subtle difference supported the idea that the homogeneous network was overfitted to the training sequence, in addition to the findings of these models’ high test error and low training error. On the other hand, the heterogeneous network could predict a larger number of sequences by each PB activity and tolerated the difference within the emotion category (Fig. 5C). For the large network, no PB activities enabled the generation of the training sequence with a sufficiently small prediction error (< 0.005) (Fig. 5D), which is consistent with the findings of the relatively large training error of these models in Fig. 3A.

### The distribution of lower-level neuron activity depending on the intrinsic heterogeneity of network excitability

Considering that many lower-level neurons showed almost constant levels of activity during sequence generation (Fig. 2B), the anatomical size of the network (total number of neurons) and the number of neurons actually recruited to embed the dynamics of facial expression (functional size of network) could be dissociated. To investigate the relationship between the functional size of the network and the intrinsic heterogeneity of network excitability, the distributions of the range of activity (i.e., the difference between the maximum and minimum outputs) were plotted as a function of the activity threshold of each lower-level neuron in the homogeneous and heterogeneous network conditions (Fig. 5E). In excessively and modestly homogenous networks, the activity thresholds are tightly distributed and the range of activity is widely distributed in non-zero values, indicating that all of the lower-level neurons are activated. On the other hand, the heterogeneous network had a wider distribution of activity thresholds, and the activity ranges of a substantial number of lower-level neurons were nearly zero. Therefore, compared with the homogeneous network, the heterogeneous network was characterized by not only the large variance in activity thresholds but also the smaller size of the functional network.

## Discussion

To the best of our knowledge, the current study is the first to evaluate facial emotion recognition based on a predictive processing framework related to ASD. The current study succeeded in showing the following: First, the perceptual categories of emotions can emerge in a hierarchical neural system through a learning process based on precision-weighted prediction error minimization. Second, the cognitive process to estimate the emotion of unseen facial expressions can be understood as the process of adjusting higher-level neural states based on minimizing precision-weighted prediction error. Third, altered facial emotion recognition in ASD can be simulated by homogeneous intrinsic neural excitability in lower-level neurons.

Using a hierarchical predictive processing framework, we demonstrated that predictive learning of facial features is sufficient for the self-organization of emotional categories in the higher level of network hierarchy without explicit emotional labels provided. Related to this finding, a previous study reported that self-organized higher-level neural representation can be used to discriminate genuine and fake emotions from facial movies using a hierarchical RNN with PB^34^. Combined with our findings, this suggests that the extraction of abstract information from dynamic facial expressions can be understood using the predictive processing framework.

**Figure 6:**
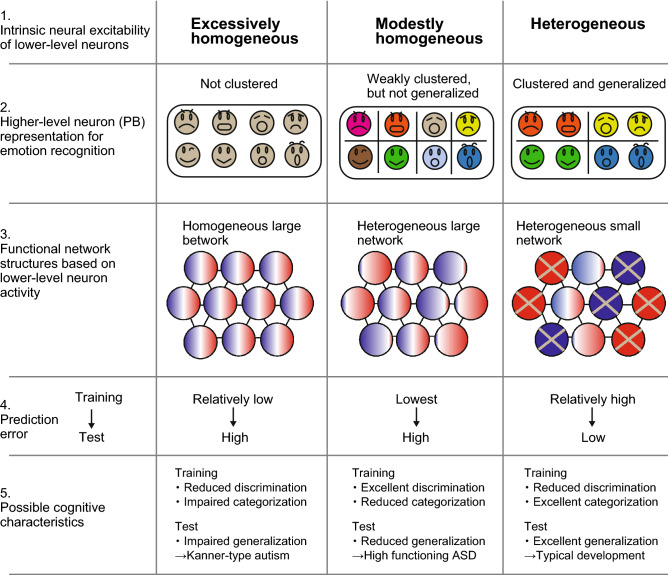
The summary of characteristics of neural networks with different neural excitability. The first row shows the names of representative network models with different intrinsic neural excitability of lower-level neurons (Table 1). The second row shows the higher-level neuron representation for emotion recognition. The 8 facial expression symbols, which can be categorized into 4 basic emotions, are shown as examples. The color represents the categorized groups by each network model. The third row shows the functional network structures in lower-level neurons. The circle represents each neuron. The color gradation in the circle represents the range of activity, and cross marks in the circle indicate that the range of activity in particular neuron is nearly zero (i.e., neurons whose activities are not changing). The fourth rows shows the prediction error in training and test datasets. The fifth rows illustrates possible cognitive characteristics and subtypes of autism spectrum disorder corresponding to each network structure.

In the current study framework, emotional categorization or recognition and generalization were influenced by the intrinsic heterogeneity of neural excitability, which could be understood by the functional size of the neural network (i.e., the number of neurons whose activity changes over time) rather than the anatomical size of the neural network (i.e., total number of neurons). The relationship between model performance and functional network structures is summarized in Fig. 6. The excessively or modestly homogeneous networks showed altered emotional recognition and impaired generalization due to an excessively high precision estimation, which is underscored by their larger functional network sizes compared with the heterogeneous (typical development) network. The comparison between excessively and modestly homogeneous networks also provides interesting insights into the subtypes of ASD. The excessively homogeneous network models could be described as defects in concept acquisition as well as severe generalization deficits, which are analogous to Kanner-type autism (traditional infantile autism). On the other hand, a modestly homogeneous network could be characterized as having the lowest training error (i.e., excellent discrimination ability based on overfitting to the details in training sequences), in addition to modest generalization deficits, which is analogous to high-functioning ASD. These differences were also attributed to the difference in functional network structure. Specifically, the distribution of the activity range was wider in the modestly homogeneous network than in the excessively homogenous network (boxplots in Fig. 5B). Therefore, considering the activity range of lower-level neurons, the functional network characteristics of the excessively and modestly homogenous networks could be described by the “heterogeneous large network” and “homogeneous large network” respectively.

The increased (anatomical) number of lower-level neurons decreased the predictive accuracy for both training and test sequences, but the emotional categories emerged after learning and could recognize the emotion of unseen sequences, which is different from characteristic ASD cognition. Indeed, there are conflicting results in studies using artificial neural networks to simulate ASD cognitive patterns by manipulating the number of neurons^35–37^. The current study suggests that, rather than anatomical network size, functional network size would be more closely related to autistic cognitive traits (i.e., altered generalization capability and perceptual emotional categorization).

Since the current neural networks model the firing frequency of a neuron population in a living brain, it is important to note that the number of neurons in our model does not directly correspond to that in the biological brain. Therefore, based on the results of the current neural network, it is difficult to directly discuss the specific number of neurons in an organism. However, we believe that we have succeeded in showing a decreasing tendency of the model’s generalization ability for excessively large functional networks. This trend, demonstrated computationally in this study, is consistent with biologically confirmed findings that ASD patients have larger brains^38^, more minicolumns^32, 33^, and an excitatory/inhibitory imbalance^39^.

We also demonstrated that mapping to normal face normalization was essential for facial emotion recognition with self-organized emotional categories in higher-level neurons. There is accumulating evidence supporting that specific brain areas (i.e., fusiform face area or superior temporal sulcus) are involved in preprocessing of facial expression information, which is different from that of the other visual objects^40–43^. Given our findings, these brain areas mediating facial information processing are likely involved in the “mapping to normal face” function to extract the emotion from the dynamic facial stimuli.

In the current study, the predictive processing framework achieved clustering by emotion using only perceptual information and also achieved generalization, but the higher-level neural representations showed overlap between PB clusters representing different emotions. This is not surprising, however, given that the higher-level neuronal representations were formed solely by predicting the visual features of facial expression images. This may reflect the fact that the visual features of facial expression images are similar, to some extent, for each emotion, and that they are not clearly divided into emotional clusters. Even in healthy subjects, confusion can occur when emotion recognition is based only on visual information of facial expressions^44^. Therefore, a clearer emotional category would be created by integrating not only facial expressions but also body posture, voice, and background information.

The spatial relationship between emotional clusters in higher-level neural representations is interesting, but there are limitations to what can be discussed from this study. In the current study, it was difficult to find a clear correspondence between the arousal and valence axes and emotional location in higher-level neural representations. One reason may be the limitation in the information that can be read from the visual information of facial images alone, as mentioned above. Another reason could be that the valence and arousal levels in the six basic emotions used in this study are not balanced, with more negative-valence and high-arousal emotions. Future studies preparing training data for such purpose could investigate the relationship between emotion-clusters and valence and arousal axes in higher-level neuron representations.

In the current study, we focused on the variance of activity thresholds in the lower-level neurons (i.e., K parameter) for simulating autistic symptoms by referring to the excitatory-inhibitory imbalance hypothesis^39^ and previous studies on network heterogeneity and efficient coding^29–31^. In this study, we achieved the best emotional clustering and generalization with K = 1000, but the optimal K value depends on other various experimental conditions^29–31^. This study was successful in demonstrating that, for a small activity threshold variance, there is a tendency for an ASD-like phenotype, but the range of K values should be considered for each experimental condition. Currently, there is a lack of biological studies available to determine the specific K value for typical development and ASD.

The current study demonstrated that the facial emotion recognition process and its alterations in ASD can be understood using a predictive processing framework based on computational psychiatry methods. Computational psychiatry methods using a predictive processing framework have been suggested to be useful in understanding autistic behavior or perception in previous studies^23, 24^, while our study is the first to suggest that these methods could also be applied to investigate the social interaction defects of ASD symptoms (i.e., affective contact). Our findings may open the door to future studies investigating the relationship between network characteristics and various components of psychiatric symptoms by simulating system-level information processing using computational psychiatry methods.

## Supplementary Information

Supplementary Figure S1.

Supplementary Figure S2.

Supplementary Methods.
