# Publisher Correction: Neural network modeling of altered facial expression recognition in autism spectrum disorders based on predictive processing framework

**DOI:** 10.1038/s41598-021-97145-2

**Published:** 2021-08-31

**Authors:** Yuta Takahashi, Shingo Murata, Hayato Idei, Hiroaki Tomita, Yuichi Yamashita

**Affiliations:** 1 Department of Psychiatry, Tohoku University Hospital, Sendai, Japan; 2 Department of Information Medicine, National Center of Neurology and Psychiatry, 4-1-1 Ogawa-Higashi, Kodaira, Tokyo 187-8502 Japan; 3 Department of Electronics and Electrical Engineering, Faculty of Science and Technology, Keio University, Tokyo, Japan; 4 Department of Intermedia Studies, Waseda University, Tokyo, Japan

Correction to: *Scientific Reports* https://doi.org/10.1038/s41598-021-94067-x, published online 26 July 2021

The original HTML version of this Article contained an error in Equation 11, where \rm was incorrectly given. The PDF version was unaffected.

$${a}_{i}\sim \backslash \text{rm N}\left(0, K\right) \qquad K = 0.001, 1, 1000,$$

now reads:

$${a}_{i}\sim \text{N}\left(0, K\right) \qquad K=0.001, 1, 1000,$$

The original Article has been corrected.

