# Supplementary material for: Neural network modeling of altered facial expression recognition in autism spectrum disorders based on predictive processing framework
Source: Sci Rep. 2021 Jul 26;11:14684. doi: 10.1038/s41598-021-94067-x (PMC8313712; doi:10.1038/s41598-021-94067-x)

Supplementary Figure 2

A Prediction error in test datasets with various network structures

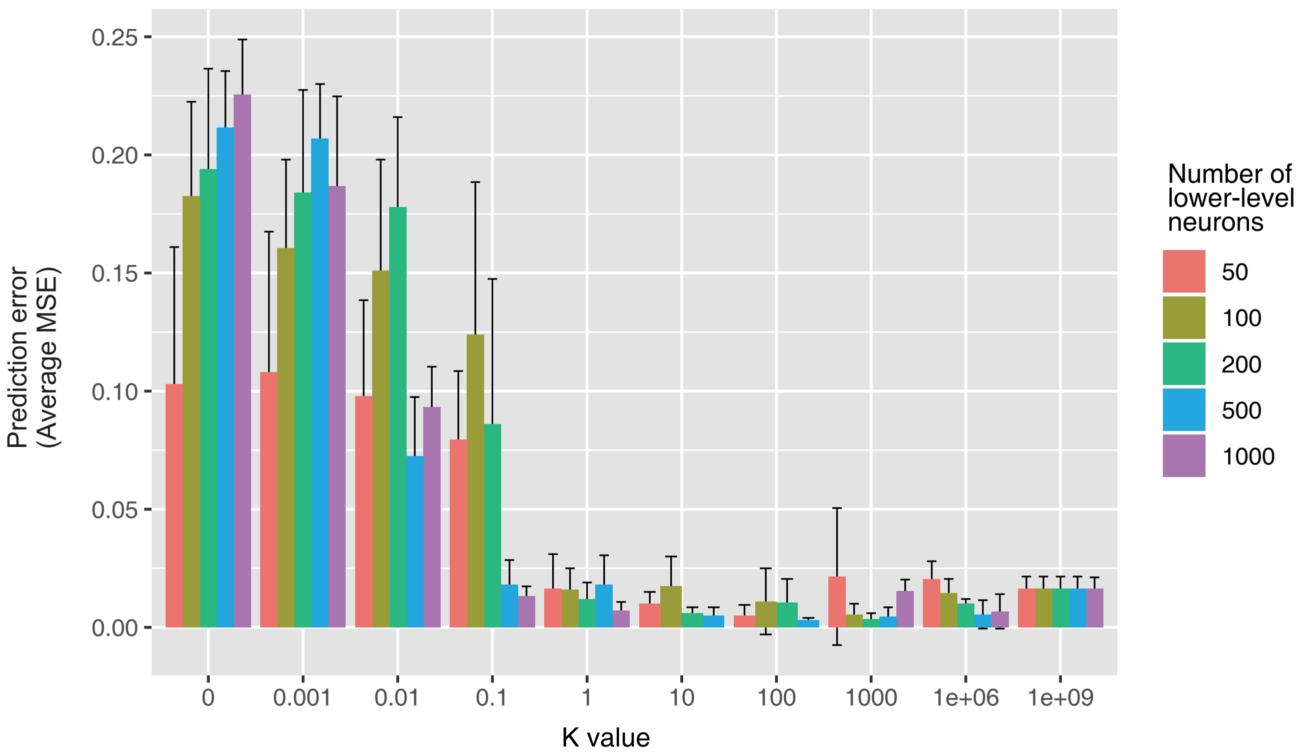

B Estimated variance with various network structures

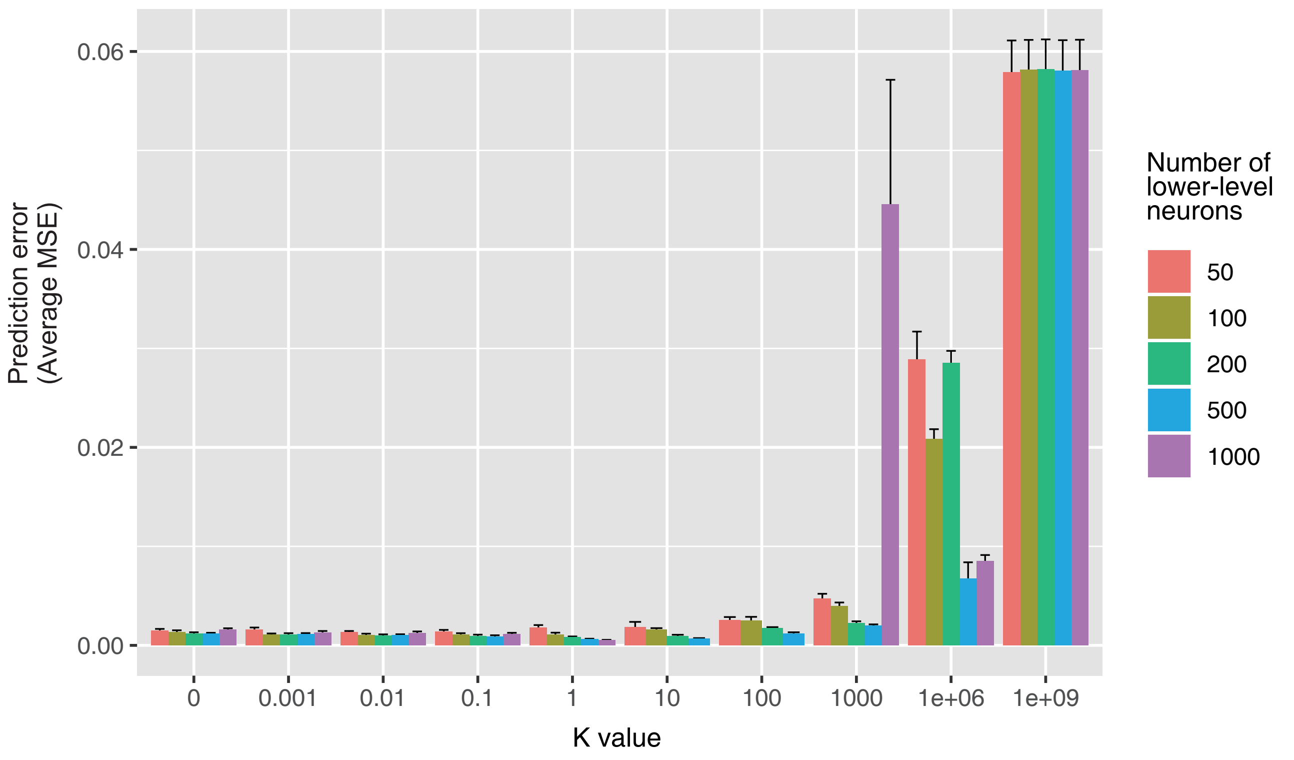

Supplement: Supplementary file 2 — Supplementary Figure S2. [file 41598_2021_94067_MOESM2_ESM.pdf]
