# Supplementary material for: Neural network modeling of altered facial expression recognition in autism spectrum disorders based on predictive processing framework
Source: Sci Rep. 2021 Jul 26;11:14684. doi: 10.1038/s41598-021-94067-x (PMC8313712; doi:10.1038/s41598-021-94067-x)
Supplement: Supplementary file 3 — Supplementary Methods. [file 41598_2021_94067_MOESM3_ESM.docx]

**Supplementary Methods**

**Selection of facial landmark features**

In this study, we analyzed the following nine features: the X-coordinate of the lip corner, and Y-coordinates of the middle of the eyebrow, the inner eyebrow, ala of the nose, the central upper lip, upper lip vermillion, lip corner, the central lower lip and lower lip vermillion, in the right face (Figure 1A). These features were selected from the X-Y coordinates of 68 facial landmarks (136 features) detected using an automated face detection and feature tracking system^1^. Owing to the limitation of computational cost, the current study, similar to previous studies^2-4^, reduced the number of features. However, there is no consensus among previous studies on which features are to be used for analysis^2-4^. Therefore, we used the following procedure to select features, as previously reported^2,3^. First, the features created from the landmarks on the left and right halves of the face were considered to be highly correlated; therefore, we excluded the 46 features (23 X-coordinates and 23 Y-coordinates) corresponding to the left half of the face. Next, we excluded 60 mostly immobile features (35 X-coordinates and 25 Y-coordinates). Finally, we eliminated 21 features (9 X-coordinates and 12 Y-coordinates) with strong correlation with other features. The remaining nine features (1 X-coordinates and 8 Y-coordinates) were used for subsequent analysis.

**Preprocessing of the sequence data**

The preprocessing of the sequence data was performed using equations (1) and (2). Suppose that we have a sequence of $\boldsymbol{x}^{\boldsymbol{(i,j)}}=(x_{1}^{\left( i,j \right)},x_{2}^{\left( i,j \right)},x_{3}^{\left( i,j \right)},\cdots,x_{T^{(i)}}^{\left( i,j \right)})$ of the $j$th feature ($1\leq j\leq9$) in the $i$th facial expression sequence ($1\leq i\leq96$), which has $T^{(i)}$time steps. First, from the vector of sequence, its first step value is subtracted to make each feature’s first step value zero.

| $\boldsymbol{x'}^{\boldsymbol{(i,j)}}=(0,x_{2}^{\left( i,j \right)}-x_{1}^{\left( i,j \right)},x_{3}^{\left( i,j \right)}-x_{1}^{\left( i,j \right)},\cdots,x_{T^{(i)}}^{\left( i,j \right)}-x_{1}^{\left( i,j \right)})$ | (1) |
| --- | --- |

Next, the values were scaled to a range of values into [-0.9, 0.9] for each feature over all target sequences.

| $MAX=max\left( {max}_{1\leq t\leq T^{(1)}}\left( {x'}_{t}^{(1,j)} \right), {max}_{1\leq t\leq T^{(2)}}\left( {x'}_{t}^{(2,j)} \right), \cdots,{max}_{1\leq t\leq T^{(96)}}\left( {x'}_{t}^{(96,j)} \right) \right)$ | (2) |
| --- | --- |
| $MIN=min\left( {min}_{1\leq t\leq T^{(1)}}\left( {x'}_{t}^{(1,j)} \right), {min}_{1\leq t\leq T^{(2)}}\left( {x'}_{t}^{(2,j)} \right), \cdots,{min}_{1\leq t\leq T^{(96)}}\left( {x'}_{t}^{(96,j)} \right) \right)$ | (3) |
| ${x''}^{\left( i,j \right)}=\frac{{x^{'}}^{\left( i,j \right)}-MIN}{MAX-MIN}\times1.8-0.9$ | (4) |

The subtraction in equation (1) was referred to as “mapping to normal face normalization” because this process unifies the positions of features in the first step among all the target sequence.

**Cross-validation**

Eight-fold cross-validation was used to evaluate the model. To avoid data imbalance for each emotion, we prepared training and test data as follows, as reported previously ^5,6^. Sixteen subjects for each of the six emotions were randomly selected from the CK+ database. These 96 participants were randomly divided into eight groups of 12 participants each, including two participants for each emotion. The analysis was then repeated with one group as test data and the remaining group as training data. The model performance was evaluated using the mean and standard error of the statistics of these eight different analyses.

**The top-down prediction process**

When predicting each target sequence, the internal state of the $i$th neuron at time step *t*, which is denoted by $u_{t,i}^{\left( s \right)}$ (t ≥ 1), was calculated as

| $u_{t,i}^{\left( s \right)}=\left\{ \begin{aligned} &u_{t-1,i}^{\left( s \right)} &i\in I_{P} \\ &\frac{1}{\tau_{i}}\left( \sum_{j\in I_{I}} w_{ij}x_{t,j}^{\left( s \right)}+\sum_{j\in I_{L}} w_{ij}l_{t-1,j}^{\left( s \right)}+\sum_{j\in I_{P}} w_{ij}p_{t,j}^{\left( s \right)}+a_{i} \right)+\left( 1-\frac{1}{\tau_{i}} \right)u_{t-1,i}^{\left( s \right)} &i\in I_{L} \\ &\sum_{j\in I_{L}} w_{ij}l_{t,j}^{\left( s \right)}+a_{i} &i\in I_{M},I_{V} \end{aligned} \right.$ | (5) |
| --- | --- |

where *I_P_*, *I_L_*, *I_I_*, *I_M_*, and *I_V_* are the index sets of the parametric bias (PB), lower-level, input, predicted mean, and estimated variance neurons, respectively; *w_ij_* is the weight of the synaptic connection from the *j*th neuron to the *i*th neuron, $x_{t,j}^{\left( s \right)}$ is the *j*th external input value at time step $t$ of the $s$th sequence, $l_{t,j}$ is the *j*th lower-level neuron activity, $p_{t,j}$ is the *j*th PB activity, $\tau_{i}$ is the time constant of the $i$th neuron, and $a_{i}$ is the activity threshold of the $i$th neuron, which determines the intrinsic neuronal excitability. From the equation above, the PB does not change in the top-down prediction process and is updated only by the bottom-up modulation process. The activity of each lower-level neuron depends on the input values, PB activities, and previous activities of itself and the other lower-level neurons. In this study, we set the initial values of the internal states of the lower-level neurons to zero, and those of the PB neurons were optimized for each target sequence through learning. This indicates that differences among multiple target sequences are represented in the activities of PB neurons and the dynamics of lower-level neuron activities. The output of each neuron was calculated using the following activation function:

| $p_{t,i}^{\left( s \right)}=\text{tanh}\left( u_{t,i}^{\left( s \right)} \right) 1\leq t\cap i\in I_{P}$ | (6) |
| --- | --- |
| $l_{t,i}^{\left( s \right)}=\text{tanh}\left( u_{t,i}^{\left( s \right)} \right) 0\leq t\cap i\in I_{L}$ | (7) |
| $y_{t,i}^{\left( s \right)}=\text{tanh}\left( u_{t,i}^{\left( s \right)} \right) 1\leq t\cap i\in I_{M}$ | (8) |
| $v_{t,i}^{\left( s \right)}=\text{exp}\left( u_{t,i}^{\left( s \right)} \right) 1\leq t\cap i\in I_{V}$ | (9) |

**Bottom-up modulation**

Bottom-up modulation is the process of parameter optimization based on precision-weighted prediction errors. This process aims to minimize the following negative log-likelihood:

| $L_{t,i}^{\left( s \right)}=\frac{\ln\left( 2\pi v_{t,i}^{\left( s \right)} \right)}{2}+\frac{\left( \hat{y}_{t,i}^{\left( s \right)}-y_{t,i}^{\left( s \right)} \right)^{2}}{2v_{t,i}^{\left( s \right)}}$ | (10) |
| --- | --- |

Here, $\hat{y}_{t,i}^{\left( s \right)}$ is the target input value of the *i*th input neuron. Minimizing this negative log-likelihood can be regarded as minimizing the precision-weighted (inverse variance-weighted) prediction error. Therefore, in this study, the negative log-likelihood is referred to as the precision-weighted prediction error.

During learning, parameters such as synaptic weights, activity thresholds of mean and variance neurons, and initial states of PB neurons are updated, and the activity thresholds of lower-level neurons are not updated to fix the distribution. Parameter optimization is performed by minimizing the sum of the negative log-likelihood over all feature dimensions, time steps, and sequences as follows:

| $L=\sum_{s\in I_{s}} \sum_{t=1}^{T^{\left( s \right)}} \sum_{i\in I_{M}} L_{t,i}^{\left( s \right)},$ | (11) |
| --- | --- |

where $I_{s}$ and $T^{\left( s \right)}$ indicate the index set and length of the $s$th target sequence, respectively. The partial derivative of each parameter, $\left( \partial L \right)/\left( \partial\theta\right)$, can be solved using the back-propagation through-time method ^7^.

In both the training and test phases, the parameters permitted to be optimized are collected by $\theta$, and $\theta$ at the $n$th iteration is updated by gradient descent on the accumulated negative log-likelihood $L$:

| $\theta\left( n \right)=\theta\left( n-1 \right)+\Delta\theta\left( n \right)$ | (12) |
| --- | --- |
| $\Delta\theta\left( n \right)=-\alpha\frac{\partial L}{\partial\theta}+\eta\Delta\theta\left( n-1 \right)$ | (13) |

Here, $\alpha$ is the learning rate and $\eta$ is a coefficient representing the momentum term. In the current study, $\alpha$ and $\eta$ were set to 0.0001 and 0.9, respectively.

The training process optimizes synaptic weights, activity thresholds of mean and variance neurons, and PB activities through 1,000,000 iterations, while the test process optimizes only PB activities through 100 iterations. The network was trained to predict future input values with a time delay ζ (in the experiment ζ =3) by receiving the input state $x_{t,i}$ at the current time step t. Therefore, the target value in equation (10) satisfies $\hat{y}_{t,i}=x_{t+\zeta,i}$.

**The evaluation of clustering of PB activities**

The current study evaluated the clustering of PB activities according to emotions by two stages. First, in the training phase, the clustering of PB activities was investigated to evaluate the emergence of emotional categories. Second, in the test phase, the similarity of a PB activity for a test sequence to the PB clusters for the training sequences of a particular emotion category was evaluated to investigate the emotion recognition process. The clustering of PB activities was evaluated by the silhouette width, which is a measure of the similarity of an object to its own cluster compared to other clusters ^8^. The silhouette width was calculated based on the similarity of a PB activity for training or test sequence to the PB clustering of training sequences with the same emotion compared to those with the other emotion.

**Heterogeneity of intrinsic neuronal excitability**

The activity threshold of the lower-level neuron, represented by $a_{i}$ ($i\in I_{L}$) in equation (5), was initialized to follow a Gaussian distribution, as shown in equation (14), and the distribution was fixed without updating the activity threshold during training. The K parameter in equation (14) represents the heterogeneity of intrinsic neuronal excitability; we investigated the relationship between the K parameter and ASD-like cognition observed in the models.

| $a_{i}\sim N\left( 0, K \right)$ $K=0.001, 1, 1000$ | (14) |
| --- | --- |

We focused on the K parameter referring to the following computational simulation and biological studies. Several simulation studies have shown that a heterogeneous neural network with a certain degree of variance in the activity threshold of neurons is necessary for efficient coding^6-8^. Furthermore, accumulating biological studies indicate an imbalance between excitatory and inhibitory signaling in patients with ASD ^5^. Recently, attempts have been made to implement such excitatory inhibitory imbalance in neural networks to reproduce ASD symptoms ^17,18^, but these previous research did not investigate emotion recognition, a core symptom of ASD. Therefore, we aimed to simulate emotion recognition from facial expressions in ASD by modulating the K parameter value.

**Number of lower-level neuron**

We also used a setting that increases the number of lower-level neuron to simulate the cognition of ASD. It has been shown in meta-analyses that children with ASD have larger brains^2^, and it has also been reported that ASD children have a higher number of minicolumns^3,4^, which is the smallest unit of neural function. Further, previous computational studies attempting to reproduce cognition in ASD have also taken the approach of increasing the number of neurons^19-21^.

References

1 Lucey, P. *et al.* in *2010 ieee computer society conference on computer vision and pattern recognition-workshops.* 94-101 (IEEE).

2 Loconsole, C., Miranda, C., Augusto, G., Frisoli, A. & Orvalho, V. *Real-Time Emotion Recognition: a Novel Method for Geometrical Facial Features Extraction*. Vol. 1 (2013).

3 Kim, Y.-G. & Huynh, X.-P. in *Computer Vision Workshop (ICCVW), 2017 IEEE International Conference on.* 3065-3072 (IEEE).

4 Horii, T., Nagai, Y. & Asada, M. Modeling development of multimodal emotion perception guided by tactile dominance and perceptual improvement. *IEEE Transactions on Cognitive and Developmental Systems* **10**, 762-775 (2018).

5 Lopes, A. T., de Aguiar, E., De Souza, A. F. & Oliveira-Santos, T. Facial expression recognition with convolutional neural networks: coping with few data and the training sample order. *Pattern Recognition* **61**, 610-628 (2017).

6 Li, L. *et al.* in *Proceedings of the 2019 International Conference on Image, Video and Signal Processing.* 85-92.

7 Rumelhart, D. E., Hinton, G. E. & Williams, R. J. Learning representations by back-propagating errors. *nature* **323**, 533-536 (1986).

8 Rousseeuw, P. J. Silhouettes - a Graphical Aid to the Interpretation and Validation of Cluster-Analysis. *Journal of Computational and Applied Mathematics* **20**, 53-65, doi:Doi 10.1016/0377-0427(87)90125-7 (1987).

9 Mejias, J. F. & Longtin, A. Optimal Heterogeneity for Coding in Spiking Neural Networks. *Physical Review Letters* **108**, 228102, doi:10.1103/PhysRevLett.108.228102 (2012).

10 Mejias, J. F. & Longtin, A. Differential effects of excitatory and inhibitory heterogeneity on the gain and asynchronous state of sparse cortical networks. *Front Comput Neurosci* **8**, 107, doi:10.3389/fncom.2014.00107 (2014).

11 Hunsberger, E., Scott, M. & Eliasmith, C. The competing benefits of noise and heterogeneity in neural coding. *Neural computation* **26**, 1600-1623, doi:10.1162/NECO_a_00621 (2014).

12 Uzunova, G., Pallanti, S. & Hollander, E. Excitatory/inhibitory imbalance in autism spectrum disorders: Implications for interventions and therapeutics. *The World Journal of Biological Psychiatry* **17**, 174-186, doi:10.3109/15622975.2015.1085597 (2016).

13 Idei, H., Murata, S., Yamashita, Y. & Ogata, T. Homogeneous Intrinsic Neuronal Excitability Induces Overfitting to Sensory Noise: A Robot Model of Neurodevelopmental Disorder. *Front Psychiatry* **11**, 762, doi:10.3389/fpsyt.2020.00762 (2020).

14 Nagai, Y., Moriwaki, T. & Asada, M. in *CogSci.*

15 Redcay, E. & Courchesne, E. When is the brain enlarged in autism? A meta-analysis of all brain size reports. *Biol Psychiatry* **58**, 1-9, doi:10.1016/j.biopsych.2005.03.026 (2005).

16 Casanova, M. F. *et al.* Minicolumnar abnormalities in autism. *Acta neuropathologica* **112**, 287 (2006).

17 Casanova, M. F., Buxhoeveden, D. P., Switala, A. E. & Roy, E. Minicolumnar pathology in autism. *Neurology* **58**, 428-432 (2002).

18 Cohen, I. L. An artificial neural network analogue of learning in autism. *Biol Psychiatry* **36**, 5-20, doi:10.1016/0006-3223(94)90057-4 (1994).

19 Cohen, I., Stein, D. & Ludick, J. Neural network analysis of learning in autism. *Neural networks and psychopathology*, 274-315 (1998).

20 Dovgopoly, A. & Mercado, E. A connectionist model of category learning by individuals with high-functioning autism spectrum disorder. *Cognitive, Affective, & Behavioral Neuroscience* **13**, 371-389 (2013).
